# A novel methodology and new concept of SARS-CoV-2 elimination in heating and ventilating air conditioning systems using waste heat recovery

**DOI:** 10.1063/5.0021575

**Published:** 2020-08-04

**Authors:** Naser Rezaei, Moharram Jafari, Ata Nazari, Sina Salehi, Faramarz Talati, Reza Torab, Rahim Nejad-Rahim

**Affiliations:** 1 University of Tabriz, Department of Mechanical Engineering, Tabriz, Iran; 2 Tabriz University of Medical Sciences, Department of Orthopedics, Tabriz, Iran; 3 Urmia University of Medical Sciences, Department of Dermatology and Infectious Diseases, Urmia, Iran

## Abstract

Heating and ventilation air conditioning systems in hospitals (cleanroom HVAC systems) are used to control the transmission/spreading of airborne diseases such as COVID-19. Air exiting from these systems may contribute to the spreading of coronavirus droplets outside of hospitals. Some research studies indicate that the shortest time of survival of SARS-CoV-2 in aerosol form (as droplets in the air) is four hours and the virus becomes inactive above 60 °C air temperature. Therefore, SARS-CoV-2 droplets cannot exit from the exhaust duct if the temperature is above 60 °C. At the condenser, heat is dissipated in the form of hot air which could be utilized to warm the exhaust air. The objective of this paper is to establish a novel technique for eliminating SARS-CoV-2 from cleanroom HVAC systems using the recovered heat of exhaust air. This can eliminate SARS-CoV-2 and reduce the greenhouse effect.

## I. INTRODUCTION

The new coronavirus (SARS-CoV-2) has triggered a global pandemic called COVID-19. The disease has been spreading within communities around the world. Researchers have been seeking solutions on how to separate SARS-CoV-2 from the communities.^1–3^ Cleanroom HVAC systems are one of the best answers to accomplish this goal. Recirculating air in ventilation systems of hospitals with infected patients can contribute to virus spreading. Propagation of the SARS-CoV-2 aerosol depends on the characteristics of HVAC system settings and air circuits. Air exiting from the cleanroom HVAC systems can spread the aerosol of SARS-CoV-2 outside of hospitals. According to scientists, the expelled droplets can travel several feet and stay suspended in the air for up to 15 min at ambient temperature.^4–6^ However, the WHO recommends the use of HEPA air filters in hospitals, infection control clinics, and other healthcare facilities to eliminate microbes and other dangerous particles. However, the efficiency of these filters depends on particle size. In other words, SARS-CoV-2 can pass through the HEPA filters, and their propagation, outside of hospitals, is unavoidable. It is also important to understand that HEPA filters do not actively kill living organisms. They capture and hold them within the matrix of the filter.^7–11^

A comprehensive review of the SARS coronavirus viability in HVAC system ventilation was conducted by Chan *et al.*^12^ and Farnsworth *et al.*^13^ The effects of temperature and relative humidity on coronavirus were analyzed by Casanova *et al.*^14^ It is found that the main criteria for coronavirus elimination are higher temperature and lower relative humidity conditions. The review paper of Mittal *et al.*^6^ stated that by increasing the temperature, the survivability of SARS-CoV-2 viruses seems to be nullified. Their results also stated the same. They concluded that higher temperature and lower relative humidity lead to larger evaporation rates that decrease the number of live viruses.

Kim *et al.*^15^ also studied the effects of humidity and other factors on the formation of a coronavirus aerosol. Ijaz *et al.*^16^ experimentally studied the relative humidity of an indoor air condition subjected to the infectious virus using an aerosolizing method. The results of the work proved that the virus survival rate depends on the relative humidity and the temperature. They found that high temperatures lead to significant coronavirus inactivation.

Correia *et al.*^4^ analyzed the ventilation systems and the airborne route of SARS-CoV-2. They proposed hypotheses on how HVAC systems can contribute to virus transmission. It was shown that HVAC systems when not appropriately used may contribute to the spreading of SARS-CoV-2.

In cleanroom HVAC systems, SARS-CoV-2 can be eliminated completely by warming the exhaust air before filtration. Cleanroom HVAC systems are also a potential source for heat recovery applications. The main purpose of heat recovery is to eliminate SARS-CoV-2. This wasted thermal energy can be transformed to electrical energy.^17^

Ramadan *et al.*^18^ used the hot air released at the condenser of an HVAC system to preheat domestic water. They found that the water temperature can increase from 25 °C to 70 °C. Ramadan *et al.*^19^ extended the work of Khaled and Ramadan^20^ to investigate reutilization of both the waste heat of the condenser and cold exhaust air. In this work, we propose an advanced cleanroom HVAC system that can eliminate coronavirus from ventilation systems by using the recovered heat of exhaust air.

The presented review of the different research works dedicated to investigating cleanroom HVAC systems shows that in most applications, outdoor air quality is not considered. In the frame of this paper, an innovative design of a clean room HVAC system is suggested. The concept relies on using the hot air of the condenser of an HVAC system to clean the exhaust air.

## II. KEY ISSUE OF CLEANROOM AIR CONDITIONING SYSTEMS IN SARS-COV-2 SITUATION

Cleanrooms often have special requirements for dry bulb temperature (DBT), relative humidity (RH), and particle concentrations. In addition, laminar ventilation is the primary mechanism for maintaining acceptable air quality in cleanrooms. In conventional designs, the supply air is cooled down to the dew point temperature for dehumidification and then reheated to achieve the desired temperature in cleanrooms. A high supply airflow rate is often required in cleanrooms for removing airborne particle pollutants. Besides removing pollutants, the three mentioned parameters may also cause SARS-CoV-2 viability for hours. To solve the problem of SARS-CoV-2 droplets exiting from HVAC systems, a key issue should be addressed properly at the design stage: it is essential to design the heat exchanger to warm the exiting air so that the viability of SARS-CoV-2 can be limited.

## III. SYSTEM DESCRIPTION

Ventilation is a primary infectious disease control strategy in hospitals and other facilities. It promotes the dilution of cleanroom air and the removal of infectious particles. However, factors related to HVAC systems in cleanrooms play an important role in airborne pathogen transmission. Spreading of the disease among healthy persons may be facilitated by this transmission.

In the current outbreak, the neighboring buildings of SARS-CoV-2 hospitals are a study case for the propagation of the disease. The main route for transmission is considered to be aerosol transmission via the central air supply or drainage systems, but obviously, other routes should not be neglected such as person-to-person transmission^21^ and touching of surfaces.^22^

In this work, we focus on the transmission of SARS-CoV-2 by aerosols during the extraction of air viral particles from cleanroom HVAC systems. In fact, as of May 1, 2020, more than 390 cases have been diagnosed in 12 locations around hospitals, including 30 deaths. These high values support our airborne transmission hypothesis.

**FIG. 1.**
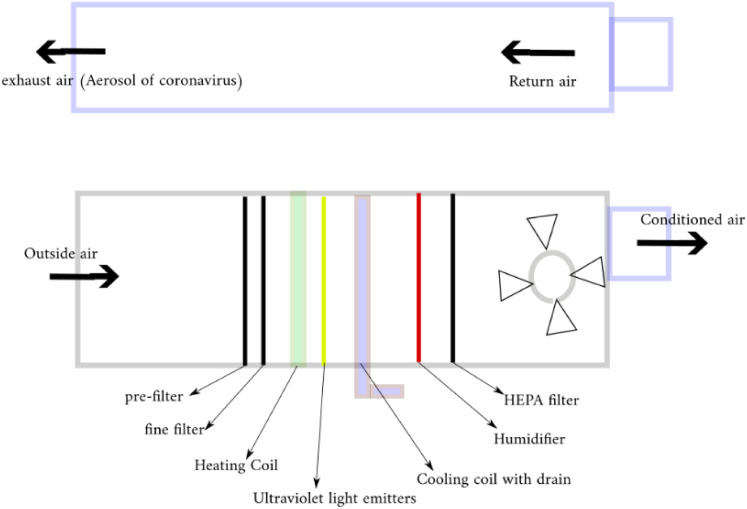
Schematic diagram of a common cleanroom HVAC system.

The schematic diagram of a common cleanroom HVAC system is presented in Fig. 1. This system consists of three main components, namely, an outdoor air intake and air exhaust ducts and controls, an air handling unit (AHU), and air distribution systems. An air handling unit by itself is composed of an HEPA filter, a humidifier, a cooling/heating coil, and ultraviolet light emitters. Figure 1 depicts the schematic diagram of an AHU.

**FIG. 2.**
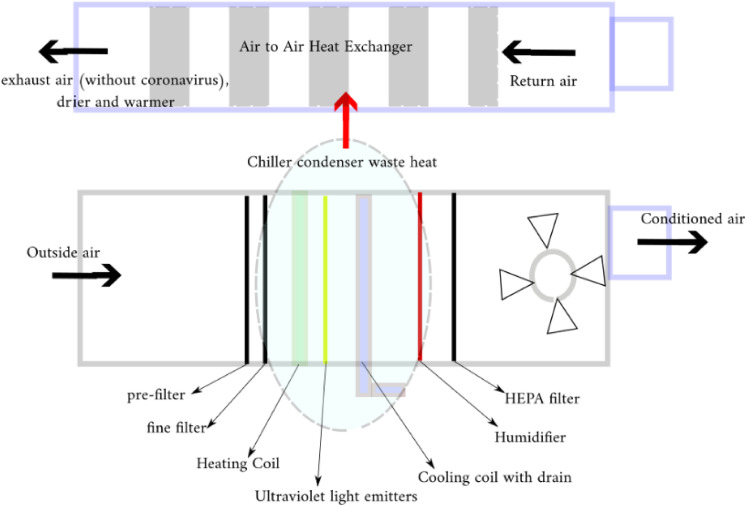
Schematic diagram of the proposed cleanroom HVAC system.

Some research studies indicate that exposing SARS-CoV-2 to a temperature of 60 °C for 5 min reduces the viral concentration by more than 99%.^23,24^ At that temperature, the study concluded that protein is a crucial component of the virus’s structure. The heat sanitizing process is carried out inside the longitudinal air to air heat exchanger, as shown in Fig. 2.

The schematic diagram of the proposed clean room HVAC system under study is depicted in Fig. 2. According to this figure, the only difference between a common cleanroom HVAC system and the proposed system is applying a longitudinal air to air heat exchanger to the waste heat recovery of the chiller condenser. With the proposed cleanroom system continuously operating at its optimum efficiency, the longitudinal air to air heat exchanger (LAIAHE), which has a long channel for heat transfer, warms the exhausted air. Therefore, SARS-CoV-2 aerosols are eliminated by this method.

**FIG. 3.**
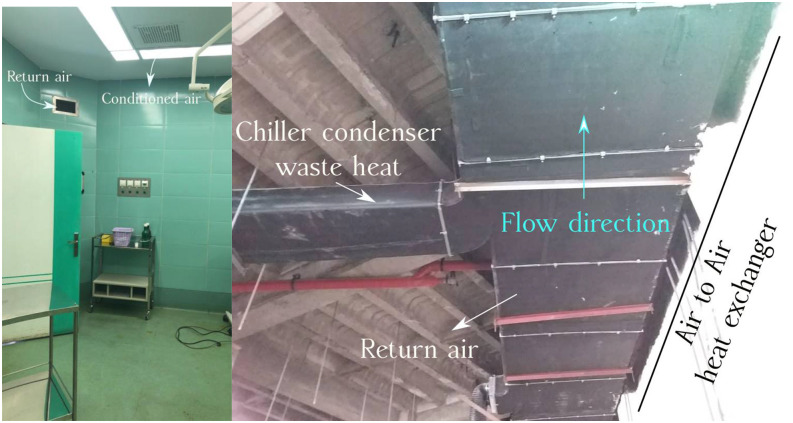
Designed clean room HVAC system [4.0 × 3.6 × 3.0 m^3^ (L × W × H)] used for the experiment, including the installed longitudinal air to air heat exchanger and ducts.

To test the effectiveness of this approach, we equipped a cleanroom with a longitudinal air to air heat exchanger, as shown in Fig. 3. We want to study the ability of the heat exchanger to destroy SARS-CoV-2. The maximum air change rate (ACR) in the designed cleanroom is equal to 23 h^−1^ (520 m^3^/h). Figure 3 presents the view of the experimental setup, including the return air duct, the chiller condenser waste heat duct, and the longitudinal air to air heat exchanger (LAIAHE). In order to generate an effective heat transfer, we have used a copper plate inside the longitudinal air to air heat exchanger. The values of the return air duct dimensions are L = 20 m, W = 1.2 m, and H = 0.6 m. In addition, the values of the heat recovery duct dimensions are L = 10 m, W = 0.4 m, and H = 0.6 m.

## IV. TEMPERATURE AND RELATIVE HUMIDITY IN THE OUTLET SIDE OF THE PROPOSED LONGITUDINAL AIR TO AIR HEAT EXCHANGER (LAIAHE)

In the experimental setup, there are two main flow rates that need to be measured: the hot air flow passing through the exchanger and the air flow passing through the exhaust channel. After measuring the velocity at different locations of the outlet of the mentioned channels, the flow rates passing through the exchanger ${\dot{\text{m}}}_{1}$ and through the duct ${\dot{\text{m}}}_{2}$ can be defined as follows:

$${\dot{m}}_{1}=\rho A_{H,Rec}V_{H,Rec},$$ (1)

$${\dot{m}}_{2}=\rho A_{exit}V_{exit}.$$ (2)

The efficiency of the proposed unit heat exchanger is calculated using the energy balance method. It is defined as the actual heat transfer divided by the maximum possible heat transfer. Assuming that there are no leakage flow, no heat loss, and no phase change, the enthalpy differences across the supply and exhaust air streams are equal. Hence, the heat transfer can be expressed as

$$\varepsilon =\frac{\dot{Q}}{{\dot{Q}}_{max}},$$ (3)

$$\dot{Q}={\dot{m}}_{1}c_{p}\left(h_{1,out}-h_{1,in}\right)={\dot{m}}_{2}c_{p}\left(h_{2,in}-h_{2,out}\right).$$ (4)

The maximum heat exchange is given by the product of the lower capacity flow and the inlet temperature difference,

$${\dot{m}}_{1}c_{p}\left(h_{1,out}-h_{1,in}\right),$$ (5)

Where ${Ċ}_{\min}$ is the lower capacity rate given by ${Ċ}_{\min}=\text{min}\left({\dot{m}}_{1}c_{p},{\dot{m}}_{2}c_{p}\right)$. Substituting Eq. (5) and Eq. (4) into Eq. (3), the exchanger heat transfer effectiveness can be computed as

$$\varepsilon =\frac{{\dot{m}}_{1}c_{p}\left(h_{1,out}-h_{1,in}\right)}{{Ċ}_{\min}\left(h_{2,in}-h_{1,in}\right)}\text{ or }\varepsilon =\frac{{\dot{m}}_{2}c_{p}\left(h_{2,in}-h_{2,out}\right)}{{Ċ}_{\min}\left(h_{2,in}-h_{1,in}\right)}.$$ (6)

The outlet temperature of the recovery heat fluid can be calculated using

$$h_{1,out}=h_{1,in}+\varepsilon \frac{{Ċ}_{\min}}{{\dot{m}}_{1}c_{p}}\left(h_{2,in}-h_{1,in}\right).$$ (7)

The heat transferred then becomes

$$Q={\dot{m}}_{1}c_{p}\left(h_{1,out}-h_{1,in}\right).$$ (8)

The outlet temperature of fluid 2 is

$$h_{2,out}=h_{2,in}-\frac{\dot{Q}}{{\dot{m}}_{2}c_{p}}.$$ (9)

Using the calculated temperature, we find the relative humidity of the exhausted air using a psychrometric chart. The temperature and relative humidity limits of the exiting air are reported to be in the range of 50 °C–80 °C and 40%–50%, respectively. The study can conclude that under such conditions, SARS-CoV-2 began disappearing rapidly.

## V. CONCLUSION

When a widespread epidemic happens, the velocity of the epidemic is one of the important parameters determining its impact on communities around the world. The rapid spread of the disease will saturate the capacity of hospitals, which in turn would lead to an increase in the mortality rate; in contrast, slower propagation would allow us more time and better resource utilization for adequate preparation. Thus, our efforts for controlling the outbreak of SARS-CoV-2 are of great importance. The proposed HVAC system can be useful in reducing the epidemic velocity. Some conclusions about the features of the proposed clean room HVAC system are summarized as follows:

1. The installation of the proposed cleanroom HVAC system is very simple.
2. The system produces exhaust air with a temperature range of 50 °C–80 °C and a relative humidity range of 40%–50%, conditions under which SAR-CoV-2 was observed to rapidly disappear.
3. Additionally, drier and warmer air can be converted to electrical energy and used to dry clothes as well.

## DATA AVAILABILITY

The data that support the findings of this study are available from the corresponding author upon reasonable request.
